# Effects of Nrf-2/HO-1, NF-κB/Cox-2/TLR-4, and Bax/Bcl-2/caspase-3 pathways in alleviating azithromycin-induced cardiotoxicity in rats: Potential cardioprotective role of crocin

**DOI:** 10.22038/ijbms.2025.83518.18069

**Published:** 2025

**Authors:** Tuba Dogan, Betul Apaydin Yildirim, Kubra Asena Terim Kapakin, Metin Kiliclioglu, Omercan Alat

**Affiliations:** 1 Department of Biochemistry, Faculty of Veterinary Medicine, Ataturk University, Erzurum, Turkey; 2 Department of Pathology, Faculty of Veterinary Medicine, Ataturk University, Erzurum, Turkey

**Keywords:** Apoptosis.Azithromycin, Cardiotoxicity, Crocin, Inflammation, Oxidative stress

## Abstract

**Objective(s):**

This study aimed to investigate the potential protective effects of crocin against azithromycin (AZ)-induced cardiotoxicity.

**Materials and Methods:**

The experimental design consisted of four groups: Control, crocin, Azithromycin, and crocin plus Azithromycin (AZ+CR 50). To evaluate oxidative stress, inflammation, and apoptosis in cardiac tissue, a combination of biochemical, molecular, and histological techniques was employed. Biomarkers such as superoxide dismutase (SOD), catalase (CAT), glutathione (GSH), nuclear factor erythroid 2–related factor 2 (Nrf-2), and heme oxygenase-1 (HO-1) were assessed to determine anti-oxidant status, while malondialdehyde (MDA) and advanced oxidation protein products (AOPP) were measured as indicators of oxidative damage. Protein levels of inflammatory markers NF-κB and toll-like receptor 4 (TLR-4) and apoptotic regulators Bax, Bcl-2, and Caspase-3 were quantified.

**Results:**

Crocin treatment effectively attenuated AZ-induced oxidative stress by enhancing anti-oxidant enzyme activity and reducing MDA and AOPP levels. Furthermore, crocin significantly down-regulated the expression of NF-κB and TLR-4 proteins, indicating reduced inflammation. The proapoptotic proteins Bax and Caspase-3, which were elevated following AZ exposure, were markedly decreased by crocin co-treatment. Conversely, the reduced expression of the antiapoptotic protein Bcl-2 caused by AZ was restored by crocin. In addition, AZ administration led to increased levels of COX-2 and MAPK-3, both of which were down-regulated following crocin treatment. Histological analysis revealed that crocin reduced degenerative and necrotic changes in heart tissue caused by AZ.

**Conclusion:**

These findings suggest that crocin exerts cardioprotective effects against AZ-induced damage by modulating oxidative stress, inflammation, and apoptosis.

## Introduction

Azithromycin is a potent macrolide antibiotic for treating various bacterial infections. It has antiviral effects due to its immunomodulatory role. Today, AZ is used alone or in combination with hydroxychloroquine in the treatment protocol for COVID-19, which has affected the world population since December 2019 (1, 2). However, cardiovascular side effects associated with AZ have recently attracted attention. Unfortunately, recent studies have reported the toxicity of AZ as it produces high levels of reactive oxygen species (ROS)(2). It is also responsible for many cardiac complications, such as prolongation of the QTc interval, ventricular tachycardia, and sudden cardiac arrest, especially in patients with a history of coronary heart disease (3). Those with heart conditions, in particular, are at greater risk for Azithromycin-induced QT prolongation (2, 4). Researchers found that azithromycin induced oxidative stress, inflammation, and apoptosis in rat heart muscle (2). In another study, Azithromycin administration increased oxidative stress and the inflammatory response as demonstrated by increases in plasma malondialdehyde (MDA) and tumor necrosis factor-alpha (TNFα)(5). The toxic effects of AZ on the heart can potentially be reduced by treatment with crocin. 

Carotenoids (a natural bioactive compound) have been the subject of research for many years due to their important role in photomedicine, photobiology, and photochemistry. Crocin (CR) is a water-soluble carotenoid obtained from the stigmas of *Crocus sativus *Linne and the fruits of *Gardenia jasminoides* Ellis (6). CR has recently attracted much attention due to its comprehensive pharmacological effects, including free radical scavenging, anti-oxidant, anti-inflammatory, anti-apoptosis, antihyperlipidemic, anti-atherosclerotic, and protective effects on DNA damage (7). The study conducted by Motlagh *et al*. shows that crocin can alleviate isoprenaline-mediated myocardial toxicity in rats (8). The protective effects of CR against cardiotoxicity have been investigated in many studies, but the underlying mechanism has not yet been elucidated (9, 10). 

We hypothesized that CR has the capacity to ameliorate AZ-induced cardiotoxicity. Therefore, this study was conducted to investigate CR’s protective effects and mechanisms against AZ-induced cardiac toxicity by conducting an *in vivo* study. Our research demonstrates the clinical effects of CR in reducing AZ-induced cardiotoxicity and its effects on apoptosis and Nrf-2/HO-1 signaling pathways.

## Materials and Methods

### Chemicals

Azithromycin (250 mg/kg) was acquired from DEVA Holding (Istanbul, Turkey). Crocin was procured from Sigma-Aldrich Chemicals (St. Louis, MO, USA). 

### Experimental animals

Twenty-four male Sprague Dawley rats with live weights ranging from 250 to 300 gr were used in the study. The rats were housed under standard laboratory conditions (24±2 ^°^C, 55±5% humidity, and 12-hr light/dark cycle). The rats were provided a balanced diet and unlimited access to tap water throughout the experiment. The Atatürk University Animal Experiments Local Ethics Committee approved the experimental protocol following the established procedures (Approval No. 2024/07/178).

### Experimental design

Rats were randomly selected; each group consisted of 6 rats after ensuring their average weights were equal. The experimental protocol was created using reference doses for CR (11) and AZ (2, 12) from previous studies.

C (Control): Rats were given 0.5 ml of physiological saline orally for 8 days.

CR 50: Rats were given 0.5 ml of 50 mg/kg crocin orally for 8 days.

AZ: Rats were given 0.5 ml of 30 mg/kg Az orally for 8 days to induce cardiotoxicity.

AZ+CR 50: Rats were given 0.5 ml of 30 mg/kg Az orally for 8 days and 0.5 ml of 50 mg/kg crocin orally 30 min later.

Twenty-four hours after the last administration (day 9), rats were decapitated under xylazine (8 mg/kg) and ketamine (60 mg/kg) anesthesia. A portion of the heart tissue was taken for biochemical analysis, while the other portion was placed in 10% formaldehyde for histopathological examination.

### Measurement of malondialdehyde (MDA), reduced glutathione (GSH), and AOPP in myocardial muscle

Myocardial muscle MDA (13) and GSH contents were measured by the specified manual methods. MDA (nmol/g tissue) was measured according to the Placer method. Briefly, thiobarbituric acid was added to the tissue homogenate and boiled in a water bath, and then the resulting color was extracted with n-butanol and measured at 532 nm. 

The GSH level (nmol/g tissue) was determined using the method established by Ellman. This analysis was based on reducing the disulfide reagent to form 2-nitro-5-mercaptobenzoic acid. The absorbance of the resulting yellow color was measured spectrophotometrically at 412 nm (14).

AOPP levels (15) were measured with a Biotek ELISA Reader (Bio Tek μQuant MQX200 ELISA reader/USA). The measured AOPP concentrations were expressed as μmol/mg protein by calculating the AOPP level in brain tissue with Chloramine-T using an extinction coefficient of 261 mmol.

### Measurement of glutathione peroxidase (GPx), superoxide dismutase (SOD), and catalase (CAT) activity in myocardial muscle

Heart tissues stored at -80 ^°^C were pulverized using liquid nitrogen and weighed to 0.5 g. The weighed heart tissues were homogenized with 0.1 ml of phosphate buffer (pH 7.4) using QIAGEN TissueLyser LT to obtain a 1:10 (w/v) homogenate and centrifuged at 11,000 rpm for 30 min at 4 ^°^C. The supernatant was used to measure GPx, SOD, and CAT activities. Total protein levels were determined using the Lowry method to calculate SOD and GPx enzyme activities (16). Myocardial muscle GPx activity was determined according to Rotruck (17). GPx activity was expressed in U/g tissue. Myocardial muscle SOD activity was determined according to Sun *et al*. (18). SOD activity was expressed in U/g tissue. Myocardial muscle CAT activity was measured according to Goth (19). CAT activity was expressed in U/g tissue.

### Western blot analysis

The protein content in myocardial muscle was measured using a method described in a previous study (11). To prepare the samples, 50 mg of weighed heart tissue was homogenized by RIPA lysis and centrifuged. Protein measurement was performed using the Thermo Pierce™ BCA measurement kit developed by Smith. The prepared samples were loaded into wells on a 10% SDS-PAGE Gel. After electrophoresis, the proteins were transferred to a PVDF membrane. The membrane was then blocked with 3% BSA for 1.5 hr. After blocking, it was washed 3 times with TBS-T. Then, β-tubulin (sc-47778), Bax (sc-20067), Bcl-2 (sc-7382), Caspase-3 (sc-56053), Nrf-2 (sc-365949), HO-1 (sc-390991), TLR-4 (sc-293072), NF-κB (sc-8008), COX-2 (sc-19999) and MAPK3 (Cat:AF0562) primary antibodies were added and incubated at +4 ^°^C overnight. After incubation, primary antibodies were removed and washed five times with TBS-T. The membrane was then incubated with goat anti-mouse IgG secondary antibodies (sc-2005, Santa Cruz Biotechnology, Inc., Texas, USA) for 1.5 hr. Secondary antibodies were removed and washed five times with TBS-T. Protein bands were visualized using Trident femto Western HRP Substrate (Catalog Number: GTX14698) and recorded using the Biorad Gel Doc XR+ Imaging System (Bio-Rad, Hercules, USA).

### Histopathological analysis

At the end of the experiment, tissue samples taken from the heart were fixed in 10% formaldehyde solution for 48 hr and embedded in paraffin blocks after routine tissue follow-up procedures. 4 µm thick sections were taken from each block, and the preparations prepared for histopathological examination were stained with hematoxylin-eosin (HE) and examined under a light microscope (Olympus BX 51, JAPAN). The sections were evaluated as none (-), very mild (+), mild (++), moderate (+++), and severe (++++) according to their histopathological features (20).

### Statistical analysis

Statistical significance was determined using one-way analysis of variance (ANOVA) with Tukey’s multiple range *post hoc* test, which was utilized for pairwise comparisons after verifying the normal distribution of the data. The results were expressed as the mean and standard error of the mean (mean±SEM), and *P*<0.05 was considered significant.

## Results

### Effects of azithromycin and crocin administration on MDA, GSH, and AOPP contents in myocardial muscle

**Figure 1:**
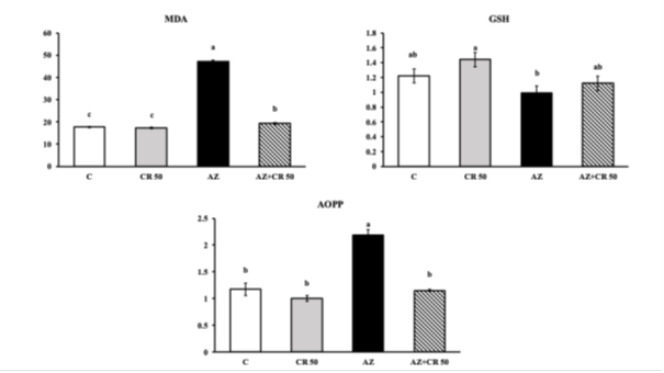
Effects of CR and AZ applications on oxidant/antioxidant levels in rat heart tissues

AZ administration significantly elevated MDA levels in cardiac tissue, rising from 17.6±1.4 nmol/mg protein in the control group to 52.4±2.3 nmol/mg protein, corresponding to a 197.7% increase (*P*<0.001). Crocin co-treatment (AZ+CR 50 group) significantly reduced MDA levels to 21.5±1.6 nmol/mg protein, reflecting a 59% decrease relative to the AZ group (*P*<0.001). AOPP levels also markedly increased in the AZ group (2.21±0.12 μmol/mg protein) compared to controls (1.14±0.08 μmol/mg protein), representing a 93.9% increase (*P*<0.001). Treatment with crocin reduced AOPP to 1.32±0.10 μmol/mg protein, a 40.3% decrease compared to AZ alone (*P*<0.001). GSH, a key endogenous anti-oxidant, was significantly reduced in the AZ group (0.91±0.07 μmol/mg protein) relative to the control (1.31±0.09 μmol/mg protein), indicating a 30.5% decline (*P*<0.001). Crocin co-treatment restored GSH levels to 1.15±0.08 μmol/mg protein, corresponding to a 26.4% increase compared to the AZ group (*P*<0.01), approaching control values. These data demonstrate that crocin effectively attenuates AZ-induced lipid and protein oxidative damage by restoring anti-oxidant balance and reducing oxidative stress markers (Figure 1).

### Effects of azithromycin and crocin administration on GPx, SOD, and CAT activity in myocardial muscle

**Figure 2:**
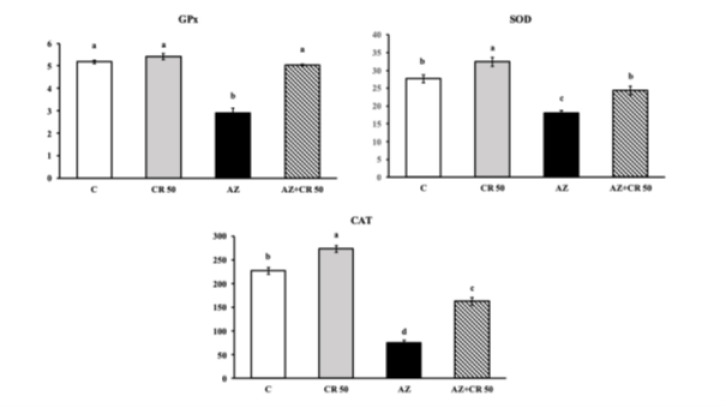
Effects of CR and AZ applications on anti-oxidant levels in rat heart tissues

As shown in Figure 2, AZ administration caused a significant reduction in the activity of key anti-oxidant enzymes compared to the control group. Superoxide dismutase (SOD) activity decreased from 32.6±1.2 U/mg protein in the control group to 14.3±0.9 U/mg protein in the AZ group, corresponding to a 56.2% decrease (*P*<0.001). Treatment with crocin in the AZ+CR 50 group significantly restored SOD activity to 26.1±1.1 U/mg protein, reflecting an 82.5% increase compared to the AZ group (*P*<0.001).

Similarly, catalase (CAT) activity was markedly reduced in the AZ group (91.7±5.4 U/mg protein) compared to the control (248.6±6.8 U/mg protein), representing a 63.1% decrease (*P*<0.001). Crocin treatment elevated CAT activity in the AZ+CR 50 group to 198.4±4.9 U/mg protein, a 116.3% increase relative to the AZ group (*P*<0.001). Glutathione peroxidase (GPx) activity followed a similar trend, decreasing from 4.7±0.3 U/mg protein in the control to 1.9±0.2 U/mg protein in the AZ group, a 59.6% reduction (*P*<0.001). Co-treatment with crocin significantly increased GPx levels to 3.8±0.2 U/mg protein, showing a 100% recovery compared to the AZ group (*P*<0.001).

### Effects of azithromycin and crocin on Bax, Bcl-2, and Caspase-3 protein levels in myocardial muscle

**Figure 3:**
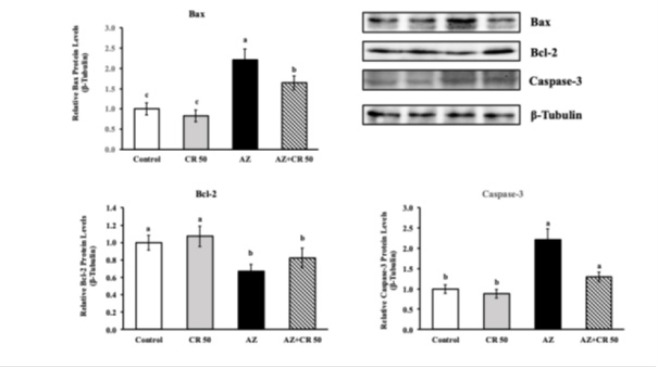
Effects of CR on AZ-induced cardiotoxicity expression levels of Bax, Bcl-2, and Caspase-3 genes in the heart

Western blot results revealed significant alterations in apoptotic marker expression following AZ administration (Figure 3). Bax, a proapoptotic protein, was significantly up-regulated in the AZ group (2.43±0.15) compared to the control (1.01±0.09), indicating a 140.6% increase (*P*<0.001). Co-treatment with crocin (AZ+CR 50) significantly reduced Bax expression to 1.34±0.12, representing a 44.9% decrease compared to the AZ group (*P*<0.001). Conversely, the antiapoptotic protein Bcl-2 was markedly reduced by AZ treatment (0.53±0.05) relative to the control group (1.22±0.08), indicating a 56.6% reduction (*P*<0.001). Crocin co-administration restored Bcl-2 levels to 1.08±0.07, showing a 103.8% increase compared to the AZ group (*P*<0.001). Similarly, Caspase-3 expression was significantly elevated in the AZ group (2.36±0.14) compared to the control (1.00±0.08), reflecting a 136% increase (*P*<0.001). Crocin treatment decreased Caspase-3 levels to 1.42±0.11, corresponding to a 39.8% reduction relative to AZ alone (*P*<0.001). These findings suggest that crocin effectively mitigates azithromycin-induced apoptosis in cardiac tissue by down-regulating proapoptotic markers and up-regulating antiapoptotic signaling.

### Effects of azithromycin and crocin on Cox-2, NF-κB, TLR-4 and MAPK-3 protein levels in myocardial muscle

**Figure 4:**
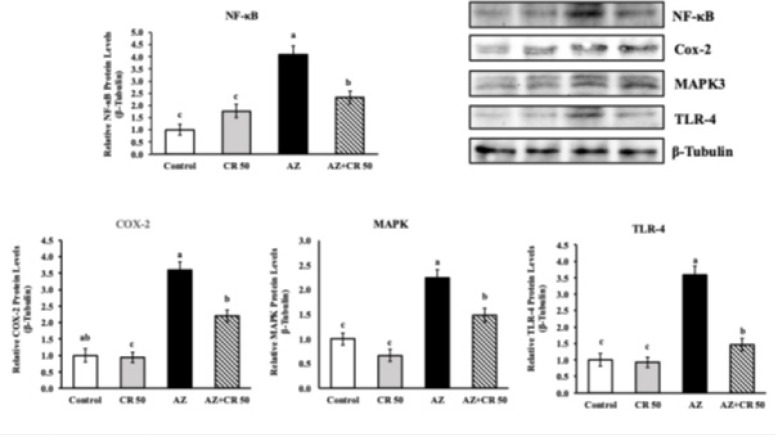
Effects of CR on AZ-induced cardiotoxicity expression levels of NF-κB, Cox-2, MAPK3, and TLR-4 genes in the heart

Western blot analysis demonstrated that AZ significantly increased the expression of major inflammatory mediators in cardiac tissue (Figure 4). The relative protein level of NF-κB rose from 1.03±0.07 in the control group to 4.62±0.22 in the AZ group, representing a 348.5% increase (*P*<0.001). Co-treatment with crocin (AZ+CR 50) markedly reduced NF-κB levels to 2.21±0.16, a 52.2% decrease compared to the AZ group (*P*<0.001). Similarly, TLR-4 expression increased from 1.09±0.08 in controls to 4.15±0.19 with AZ treatment, a 280.7% elevation (*P*<0.001), while crocin reduced this to 2.03±0.15, corresponding to a 51.1% reduction versus AZ alone (*P*<0.001).

COX-2 levels were also significantly elevated by AZ (3.86±0.18) compared to control (1.01±0.06), indicating a 282.2% increase (*P*<0.001). Crocin treatment brought COX-2 levels down to 1.89±0.12, a 51% decrease compared to AZ (*P*<0.001). Likewise, MAPK-3 levels increased from 1.05±0.07 in control rats to 2.78±0.14 in the AZ group, a 165.7% rise (*P*<0.001). This was significantly reduced to 1.48±0.11 in the AZ+CR 50 group, representing a 46.8% decline (*P*<0.001). These results clearly indicate that crocin effectively attenuates AZ-induced up-regulation of key pro-inflammatory pathways in heart tissue.

### Effects of azithromycin and crocin on HO-1 and Nrf-2 protein levels in myocardial muscle

**Figure 5:**
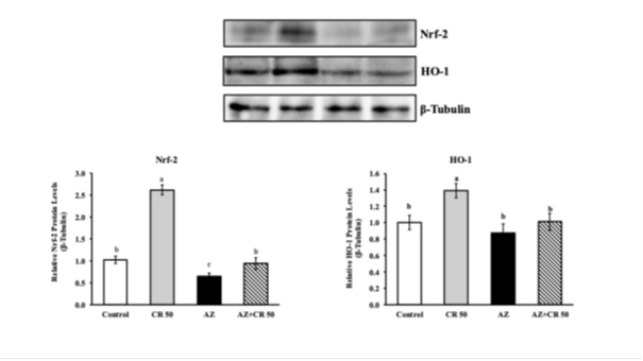
Effects of CR on AZ-induced cardiotoxicity expression levels of Nrf-2 and HO-1 genes in the heart

Western blot analysis demonstrated that AZ treatment significantly suppressed the expression of Nrf-2 and HO-1 in heart tissue (Figure 5). The relative expression of Nrf-2 decreased from 2.65±0.18 in the control group to 0.94±0.12 in the AZ group, corresponding to a 64.5% reduction (*P*<0.001). Crocin co-treatment (AZ+CR 50) elevated Nrf-2 levels to 1.98±0.14, reflecting a 110.6% increase compared to the AZ group (*P*<0.001), though still below control values. Similarly, HO-1 expression was significantly reduced from 1.32±0.09 in the control group to 0.47±0.08 in the AZ-treated rats, indicating a 64.4% decrease (*P*<0.001). Treatment with crocin markedly increased HO-1 levels in the AZ+CR 50 group to 1.01±0.07, representing a 114.9% increase relative to the AZ group (*P*<0.001). These findings suggest that crocin effectively restores AZ-induced down-regulation of the Nrf-2/HO-1 pathway, essential for anti-oxidant defense and cellular protection.

### Histopathological changes of azithromycin and crocin on myocardial muscle

**Figure 6:**
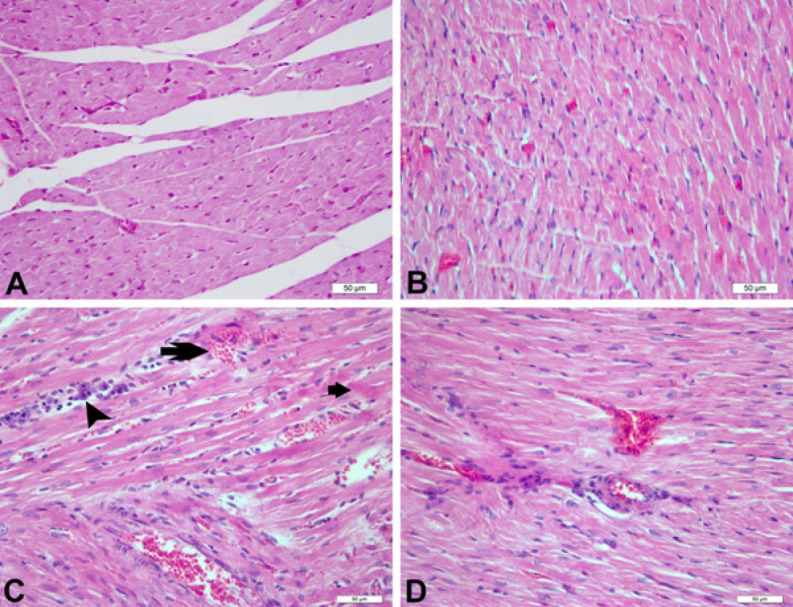
Heart tissue, histopathological findings

When the heart tissues were examined histopathologically, it was determined that the control and crocin groups had normal histological structures. The vessels were found to be hyperemic. Severe degenerative and necrotic changes were seen in the heart muscle cells. In addition, mononuclear cell infiltrates, mainly lymphocytes, were seen in the interstitial region and around the vessels. In addition, areas of hemorrhage were seen. Although similar findings were observed in the AZ+CR 50 group, degenerative and necrotic changes, and inflammatory reactions were reduced in the animals in this group (Figure 6 and Table 1).

## Discussion

Azithromycin, a second-generation macrolide, is widely used as a broad-spectrum antibiotic. Studies in rat models have shown that azithromycin induces oxidative stress, inflammation, and apoptosis in cardiac tissue. These effects can lead to ECG changes, myocardial infarction, and even death (2, 21). Crocin is a powerful anti-oxidant with lipid-lowering activity. It effectively prevents cardiovascular diseases and has strong anti-oxidative and anti-inflammatory properties (22). This study was designed to investigate the pathological changes induced by AZ in rats and the potential protective effects of crocin through biochemical and histological analyses.

Recent pharmacological investigations have demonstrated that CR holds promise as a novel therapeutic agent due to its antitumor, anti-oxidant, and free radical scavenging properties. Anti-oxidants are essential in protecting biological systems from free radical-induced damage by neutralizing reactive oxygen species (23, 24). Razavi and colleagues suggested that CRO may effectively alleviate cardiac damage due to oxidative stress (25). Although the precise mechanisms through which CRO, as a water-soluble carotenoid, exerts its free radical scavenging activity remain unclear, it is generally accepted that its mode of action resembles that of other well-characterized carotenoids (24). Therefore, it is plausible to hypothesize that CRO contributes to regulating endogenous anti-oxidant enzymes once absorbed into the bloodstream, thereby modulating intracellular oxidative stress (23, 24). It has been extensively documented that AZ can initiate ROS production, which subsequently causes oxidative stress and inflammation (5); these two important pathological factors play a role in the development and progression of AZ-induced cardiotoxicity (2). AZ increases the susceptibility of cardiomyocytes to ROS-induced oxidative attack through the depletion of the anti-oxidant defense mechanism, leading to DNA damage, lipid peroxidation, and apoptosis (26). In this study, the oxidative stress pathological pathway was activated following AZ administration, and this was detected by increased cardiac MDA and AOPP levels and a significant decrease in anti-oxidant enzyme activity, whereas treatment of animals with CR resulted in a significant decrease in cardiac malondialdehyde and AOPP levels and a corresponding increase in cardiac glutathione levels. It also significantly increased SOD, CAT, and GPx anti-oxidant enzyme activities. Previous studies have shown that phytochemical components of CR exhibit excellent anti-oxidant ability, and their effects on lipid peroxidation and anti-oxidant parameters have been highlighted.

CR reduced oxidative stress and significantly mitigated inflammation in the hearts of AZ-treated rats. AZ-induced cardiac inflammation was characterized by the up-regulation of TLR-4, NF-κB p65, and COX-2. Inflammation is a key factor in AZ-related cardiotoxicity, with a heightened inflammatory response and increased levels of pro-inflammatory mediators observed in the hearts of AZ-exposed rats (5). *In vitro* studies further confirmed ROS production in AZ-treated cardiomyocytes triggered inflammatory signaling pathways and cell death. The observed increase in TLR-4 and related inflammatory markers is likely a direct result of oxidative stress, as excessive ROS activates TLR-4, which in turn stimulates NF-κB. This activation of NF-κB leads to the release and expression of various pro-inflammatory mediators, as shown in this study (27). COX-2 expression is also triggered by tumor promoters, growth factors, and pro-inflammatory substances (28). The up-regulation of these mediators results from the activation of the TLR-4/NF-κB signaling pathway caused by AZ exposure (27). 

Furthermore, MAPKs, serine/threonine protein kinases, control cell differentiation, proliferation, survival, and death in addition to their important roles in controlling inflammatory mediators (29). They are also activated in response to ROS-induced oxidative and nitrative stress, leading to increased apoptosis. In addition to increased levels/expression of MAPK protein, cytokines, and inflammatory mediators, AZ-induced cardiac injury showed significantly increased MAPK activation (30). However, CR treatment of AZ-injected rats inhibited AZ-induced NF-κB activation, significantly inhibited the phosphorylation of MAPK signaling proteins, and attenuated inflammatory mediators. This indicates positive regulation of MAPK family proteins JNK and P38 and plausibly explains the underlying anti-inflammatory mechanisms of BSB in AZ-induced cardiotoxicity.

In addition to its well-documented anti-oxidant properties, accumulating evidence suggests that oxidative stress and the resulting generation of free radicals play a central role as apoptotic triggers in various pathological conditions, including cardiovascular diseases. Reactive oxygen species are known to induce apoptosis and cause DNA damage (31), while anti-oxidant compounds are believed to attenuate these effects significantly (32). Crocin has been shown to reduce methyl methanesulfonate (MMS)-induced DNA damage in a dose-dependent manner (33). Moreover, CR modulates apoptotic signaling pathways by inhibiting tumor necrosis factor (TNF)-induced cell death, altering the expression levels of Bcl-2 family proteins such as Bax and Bcl-2, blocking cytochrome c-induced caspase-3 activation, and ultimately preventing DNA fragmentation (33-35). These findings are consistent with previous reports indicating that anti-oxidant agents may inhibit apoptosis by regulating gene expression and intracellular signal transduction pathways. For instance, Razavi *et al*. demonstrated that CR exerts cardioprotective effects against diazinon-induced toxicity by reducing lipid peroxidation, histopathological damage, and apoptosis through decreasing the Bax/Bcl-2 ratio, inhibiting cytochrome c release, and preventing caspase-3 activation (25). The balance between Bcl-2, an antiapoptotic protein, and Bax, a proapoptotic counterpart, is crucial in maintaining mitochondrial membrane integrity and controlling the intrinsic apoptotic pathway by regulating cytochrome c release (31). AZ-induced oxidative stress activates the innate mitochondria-dependent apoptotic pathway in cardiac tissues. AZ-induced apoptotic signaling has been shown to involve several processes, including increasing the activities of Caspase-3 and Bax proteins while decreasing the activities of Bcl-2 proteins (27, 36). Consistent with previous studies, the present study observed that AZ administration led to significant cardiac apoptosis, which was observed by significant increases in the density of Caspase-3 and Bax in cardiac tissues. In addition, the density of Bcl-2 was found to be reduced. In particular, CR treatment significantly improved the treated rats by increasing the levels of Bcl-2 while decreasing the levels of Caspase-3 and Bax.

Activation of several cytoprotective factors may protect against AZ by reducing oxidative and inflammatory damage. Nuclear factor erythroid 2-related factor 2 (Nrf-2) is effective in preventing tissue damage by promoting the transcription of several cytodefensive factors, including heme oxygenase-1 (HO-1) (37). We hypothesized that Nrf-2 and HO-1 up-regulation contributes to the protective efficacy against AZ-induced cardiotoxicity. AZ decreased Nrf-2 and HO-1 levels; this effect was significantly prevented in CR-treated mice. CR up-regulated Nrf-2 and HO-1 in the hearts of AZ-treated mice; this effect was explained by the increased protective effects. Nrf-2 and HO-1 may prevent maintenance by inhibiting NF-κB, activating anti-inflammatory adducts, and attenuating oxidative damage. Direct and indirect inhibition of NF-κB via HO-1 induction. It is possible that HO-1 transcription is directly mediated by PPARγ and that upon activation, its application attenuates ROS production and apoptotic cell destruction (37, 38).

Histopathological findings of the heart pretreated with crocin presents a well preserved normal morphology of cardiac muscle with no evidence of necrosis when compared to AZ-control heart. Crocin *per se* also exhibits normal cardiac fibers without any pathological changes, therefore indicating that crocin itself does not possess any adverse effects on myocardium.

Malondialdehyde (MDA), Glutathione (GSH), and Advanced oxidation protein products (AOPP). Values are given as mean±SE

C: control rats; CR: crocin 50 mg/kg rats; AZ: Azithromycin 30 mg/kg rats; AZ+CR 50: Azithromycin 30 mg/kg and crocin 50 mg/kg treated rat

a, b, c: Significant differences between means indicated by different letters within the same column

Glutathione peroxidase (GPx). Superoxide dismutase (SOD). Catalase (CAT). Values are given as mean±SE

C: control rats; CR: crocin 50 mg/kg rats; AZ: Azithromycin 30 mg/kg rats; AZ+CR 50: Azithromycin 30 mg/kg and crocin 50 mg/kg treated rat

a, b, c, d: Significant differences between means indicated by different letters within the same column

The bar graph is shown as mean±standard error (SEM)(n=6)(One-way ANOVA and Tukey’s test) (***P<0.001)

C: control rats; CR: crocin 50 mg/kg rats; AZ; Azithromycin 30 mg/kg rats; AZ+CR 50: Azithromycin 30 mg/kg and crocin 50 mg/kg treated rat

a, b, c: Significant differences between means indicated by different letters within the same column

The bar graph is shown as mean±standard error (SEM)(n=6)(One-way ANOVA and Tukey’s test) (***P<0.001)

C: control rats; CR: crocin 50 mg/kg rats; AZ: Azithromycin 30 mg/kg rats; AZ+CR 50: Azithromycin 30 mg/kg and crocin 50 mg/kg treated rat

a, b, c: Significant differences between means indicated by different letters within the same column

The bar graph is shown as mean±standard error (SEM)(n=6)(One-way ANOVA and Tukey’s test)(***P<0.001)

C: control rats; CR: crocin 50 mg/kg rats; AZ: Azithromycin 30 mg/kg rats; AZ+CR 50: Azithromycin 30 mg/kg and crocin 50 mg/kg treated rat

a, b, c: Significant differences between means indicated by different letters within the same column

**Table 1:** Scoring of histopathological findings in heart sections

Groups	Degeneration	Necrosis	Hemorrhage	*P*-value
Control	0.40 0.24 ^c^	0.00 0.00 ^c^	0.20 0.20 ^c^	<0.001
CR 50	0.60 0.24 ^c^	0.00 0.00 ^c^	0.20 0.20 ^c^	<0.001
AZ	3.80 0.20 ^a^	2.60 0.24 ^a^	3.60 0.24 ^a^	<0.001
AZ+CR 50	1.80 0.20 ^b^	1.80 0.20 ^b^	1.60 0.24 ^b^	<0.001

Data of rats in each group were expressed as mean±standard error (SE)(n=6). The different letters (a-c) on the same line indicate a statistically significant difference (***P<0.001) between the groups

CR: Crocin, AZ: Azithromycin

Control (A), crocin (B), Azithromycin (C), Azithromycin+crocin 50 (d), hemorrhage (large arrow), necrosis in muscle cells (small arrow), inflammatory cells (arrowhead), H&E, Bar: 50µm

## Conclusion

This study concluded that azithromycin may produce oxidative stress by inhibiting free radicals and various anti-oxidant enzymes. Azithromycin may also increase inflammation and apoptosis. Therefore, it causes ischemic necrosis and thus alters various physiological and functional changes in the heart. Crocin administration showed cardioprotective effects by significantly reducing azithromycin-induced oxidative stress, which can be attributed to its anti-oxidant, anti-inflammatory, and antiapoptotic effects. Crocin has also been shown to increase anti-oxidant capacity by activating Nrf-2/HO-1 signaling pathways.

## Data Availability

Data are available upon request from the corresponding authors.

## Declaration

We have not used any Al tools or technologies to prepare this manuscript.
